# The effect of SensiKIN mouthwash on shear bond strength of orthodontic brackets on enamel surface: An in vitro study

**DOI:** 10.34172/joddd.025.41855

**Published:** 2025-03-31

**Authors:** Mehdi Daneshpooy, Parastou Nastarin, Seyyedeh Shabnam Sajjadi

**Affiliations:** ^1^Department of Operative Dentistry, Faculty of Dentistry, Tabriz University of Medical Sciences, Tabriz, Iran; ^2^Department of Orthodontics, Faculty of Dentistry, Tabriz University of Medical Sciences, Tabriz, Iran; ^3^Department of Pediatric Dentistry, Faculty of Dentistry, Tabriz University of Medical Sciences, Tabriz, Iran

**Keywords:** Orthodontics, SensiKIN mouthwash, Shear bond strength

## Abstract

**Background.:**

Mouthwashes are used to control and decrease problems such as the accumulation of microorganisms and plaque in patients undergoing orthodontic treatment. Since the success of fixed orthodontic treatment depends on the bond strength of brackets to the enamel, the present study investigated the possible effects of the SensiKIN mouthwash on the shear bond strength of orthodontic brackets on the enamel surface.

**Methods.:**

The present case–control study was carried out on 40 extracted sound human premolar teeth (20 in the mouthwash group and 20 in the control group). All the tooth samples were immersed in 0.1% thymol solution for seven days, followed by storage in distilled water at 4ºC for<3 months. In the mouthwash group, the SensiKIN mouthwash was applied to the teeth twice every day for one minute each time for one month. The teeth in both groups were retrieved from the storage solutions for orthodontic procedures. Finally, a universal testing machine was used to determine the shear bond strength of orthodontic brackets to the enamel surface. The data were analyzed using SPSS 26.

**Results.:**

The results showed no significant difference in the mean shear bond strengths of orthodontic brackets between the SensiKIN mouthwash group (117.8, 36–387 N, around 11.7 Mpa) and the control group (121.6, 40.3–473.3 N, around 12.1 MPa) (*P*=0.914).

**Conclusion.:**

Since the SensiKIN mouthwash did not decrease the shear bond strength of orthodontic brackets, it can be used during orthodontic treatment.

## Introduction

 Increasing the patients’ awareness and changes in their lifestyle have resulted in increased demands for orthodontic treatments.^1^ Brackets are one of the main components in orthodontic treatment. Comprehensive orthodontic treatment involves the enamel surfaces etching and bonding the brackets with composite resins.^2^ The success of fixed orthodontic treatments depends on the bond strength of brackets to the enamel. A minimum bond strength of 6–8 MPa is required to stabilize brackets on tooth surfaces during orthodontic treatment. If such strength decreases, the brackets will be debonded from the tooth surface, resulting in prolonged treatment and patient and orthodontist dissatisfaction.^3^

 On the other hand, since the brackets and wires are bonded to tooth surfaces, fixed orthodontic treatment is usually associated with plaque accumulation, poor oral hygiene, white spot lesions, and an increased risk of dental caries. Different tools and materials have been recommended for these patients, including special orthodontic toothbrushes, interdental brushes, special orthodontic dental floss, and mouthwashes.^4,5^ Mouthwashes used to control plaque and gingivitis have different compositions and properties, and depending on their chemical composition, they might cause changes in the tooth enamel, affecting the bond strength of brackets to the tooth surface.^6-11^

 SensiKIN is a commonly used anti-hypersensitivity mouthwash with potassium nitrate as its active ingredient. Many studies have confirmed the anti-hypersensitivity action of potassium nitrate.^12,13^ SensiKIN products contain sodium fluoride as the active ingredient in addition to potassium nitrate. Sodium fluoride increases the effect of potassium nitrate in preventing tooth sensitivity, resulting in the long-term protection of sensitive teeth. Furthermore, sodium fluoride prevents dental caries and strengthens enamel. In addition, SensiKIN mouthwash protects and regenerates gingival tissues and maintains gingival tissue consistency and oral mucous layers since it contains vitamin E and precursors of vitamin B_5_.^14^ Although mouthwashes protect against bacteria, they might affect the bond strength of orthodontic brackets. Since the success of fixed orthodontic treatment depends on the bond strength of brackets to the enamel, the present study aimed to investigate the possible effects of the SensiKIN mouthwash on the shear bond strength of orthodontic brackets on the enamel surface.

## Methods

 The present case‒control study was performed to explore the effect of SensiKIN mouthwash on the shear bond strength of orthodontic brackets to enamel surfaces. To determine the sample size, a study by Demir et al^15^ was considered for the means ± standard deviations of shear bond strength values in the control (no mouthwash) and case (SensiKIN mouthwash) groups (31.64 ± 3.62 and 36.56 ± 5.59, respectively), with type I error of α = 0.05 and a study power of 80%. The initial sample size was n = 34 (n = 17, each group). Then, to increase the study’s validity and eliminate the errors resulting from dropouts during the study, the sample size was increased by 20%, and finally, 40 samples (n = 20, each group) were included.

 The samples (sound premolar teeth) were randomly selected using the convenient sampling method from the sound premolar teeth extracted for orthodontic reasons at Tabriz Faculty of Dentistry. Teeth with hypoplastic areas, cracks, and previous treatments with chemical agents such as alcohol, formalin, and hydrogen peroxide on the tooth surface were excluded.

 The samples were randomly assigned to the groups (n = 20) of case and control. All the teeth were immersed in 0.1% thymol solution (wt/vol) for disinfection and to prevent dehydration, followed by storage in distilled water at 4 ºC for less than three months. Distilled water was refreshed every week.^16-18^

 No mouthwash was used in the control group. In the case group, SensiKIN mouthwash was applied twice daily for one minute each time for a month to the tooth samples in this group. After each application, the teeth were rinsed with deionized water.^15^

**Figure 1:**
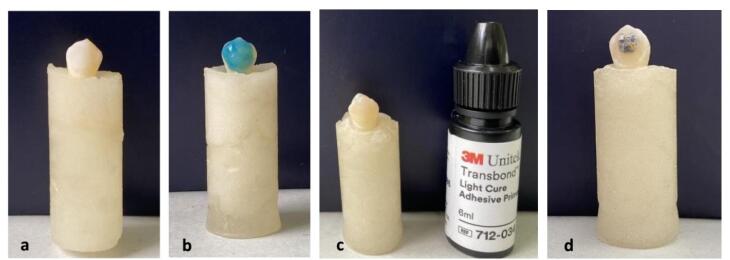
Tooth preparation steps. (a) Rinsing and drying the teeth. (b) Etching the teeth with 37% phosphoric acid. (c) Applying Transbond XT primer-adhesive to the tooth surface. (d) Bonding the brackets using Transbond XT light-cured composite resin

**Figure 2:**
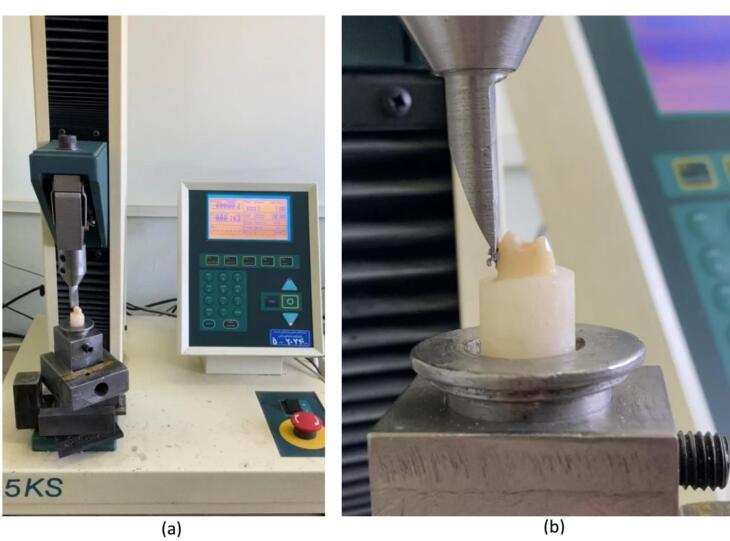
Evaluating the shear bond strength. (a) Universal testing machine. (b) The machine connected to a blade at a crosshead speed of 1 mm/min

 The teeth in both groups were retrieved from the storage solutions for orthodontic procedures. The soft tissue remnants and calculi were removed from the tooth surfaces, and the teeth were cleaned with fluoride-free pumice and a rubber cup.^15,19^ The tooth crowns were mounted in a 3-cm round mold using self-cured acrylic resin. The crowns were mounded at a right angle to the mold.^10,15^ The tooth surfaces underwent a prophylactic procedure with a rubber cup and slurry of pumice and water at low speed (3000 rpm) before the bonding procedures, followed by rinsing with water for 10 seconds and drying with an air syringe (Figure 1a).^2^ The crown’s labial surface was etched with 37% phosphoric acid (Figure 1b) for 15 seconds, and after 10 minutes of irrigation with an air syringe, it was dried with an air syringe to achieve a dry chalky surface. A thin homogeneous layer of Transbond XT primer-adhesive (Figure 1c) was applied to the tooth surface.^2,18^ The brackets were bonded to tooth surfaces using Transbond XT light-cured composite resin (Figure 1d) at the center of the inciso-gingival buccal surface on the long axis of the teeth. The brackets were firmly placed on the tooth surface so that the minimum thickness of composite resin would remain between the bracket base and the tooth surface.^18^ After removing excess composite resin with a dental explorer, the light-curing process was performed for 10 seconds from the mesial aspect and 10 seconds from the distal aspect.^18^ The samples were stored in distilled water at 37 ºC for 24 hours before the bond strength tests.^15^ To evaluate the shear bond strength, debonding was carried out using a universal testing machine (Figure 2a) connected to a blade at a crosshead speed of 1 mm/min. The bond strength values were recorded in both groups (Figure 2b).^20,21^ Finally, the shear bond strength values of orthodontic brackets to enamel surfaces were recorded in the case and control groups. The data were analyzed using SPSS 26.

## Results

**Figure 3:**
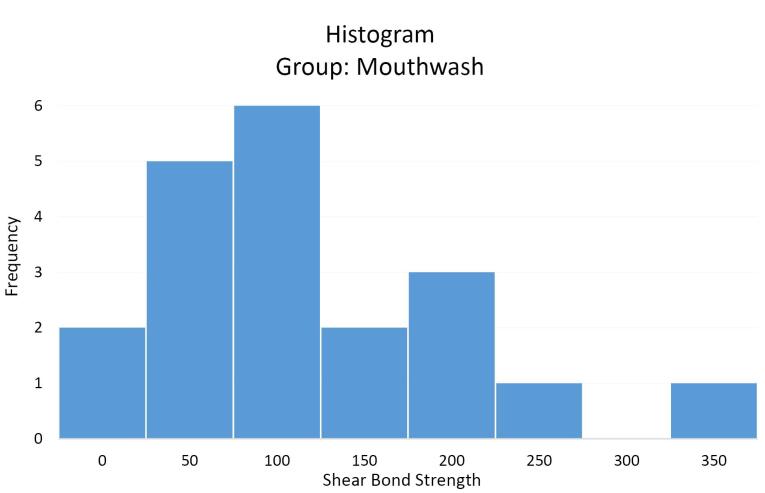
The distribution of shear bond strength values of orthodontic brackets in the case group

**Figure 4:**
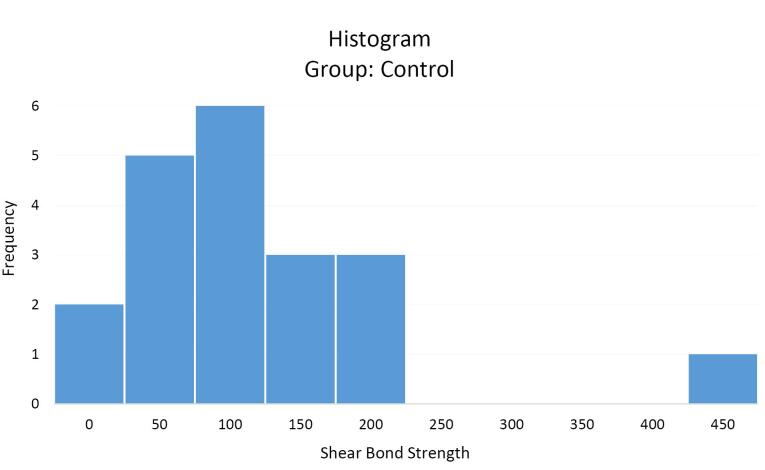
The distribution of shear bond strength values of orthodontic brackets in the control group

 The mean shear bond strength values of orthodontic brackets in the case (SensiKIN mouthwash) (Figure 3) and control (Figure 4) groups were 117.8 (36–387) and 121.6 (40.3–473.3) Newtons, respectively.

**Figure 5:**
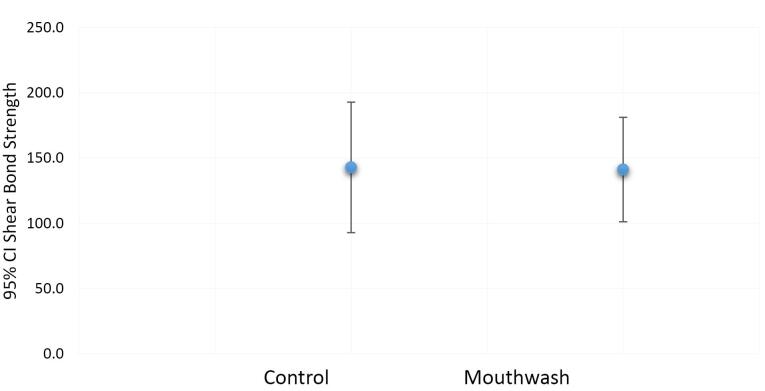
The mean shear bond strength values of orthodontic brackets in the two groups

 Mann-Whitney test showed no significant differences in the mean shear bond strength values of orthodontic brackets between the two groups (*P* = 0.914) (Figure 5).

## Discussion

 Many factors, such as dietary changes and variations in salivary flow, can affect the bond strength of the brackets during fixed orthodontic treatment.^22^ On the other hand, placing brackets increases the odds of the accumulation of microorganisms and plaque around the brackets, increasing the risk of dental caries, gingival inflammation, and enamel decalcification.^23,24^ Therefore, different methods, such as various mouthwashes, are used in orthodontic patients to manage and decrease such problems.^25,26^ Although mouthwashes protect against bacteria, different mouthwashes exert different effects on the bond strength.^9,15^ An optimal bond has a sufficiently high bond strength to resist bracket debonding during orthodontic treatment. Since decreased bond strength can increase the risk of bond failure, prolongation of treatment, the incidence of caries and periodontal diseases, and decreased patient satisfaction with orthodontic treatment, it is crucial to introduce a suitable mouthwash from the periodontal viewpoint, with no effect on the bond strength of brackets.^27^

 The present study evaluated the effect of SensiKIN mouthwash on the shear bond strength of orthodontic brackets to the enamel surface. The results showed that the shear bond strength of brackets in the SensiKIN and control groups were 117.8 N (36‒307) (around 11.7 MPa) and 121.6 N (40.3‒473.3) (around 12.1 MPa), respectively. Since the minimum bond strength for the stability of brackets on the tooth surface during treatment is approximately 6–8 MPa,^3^ the bond strength after using the SensiKIN mouthwash was slightly higher than the clinically acceptable shear bond strength.

 A study by Javanmardi et al^28^ showed that SensiKIN did not decrease the forces of elastic chains and NiTi coil springs. The present study showed no significant differences in orthodontic brackets’ mean shear bond strengths between the SensiKIN and control groups. This mouthwash did not decrease the shear bond strength of orthodontic brackets. Although no previous study has evaluated the effect of SensiKIN mouthwash on the shear bond strength of orthodontic brackets, Catalbas et al^29^ showed that the mean shear bond strength of orthodontic brackets after using chlorhexidine mouthwash was 24.9 ± 2.75 MPa, which was not different from the control group.

 Each mouthwash has its specific composition; therefore, the effects of the components and ingredients on the bond strength should be considered.^6,30^ SensiKIN mouthwash is a non-alcoholic mouthwash containing potassium nitrate, sodium fluoride, vitamin E, and vitamin B_5_ precursor.^14^ Many studies have confirmed the anti-sensitivity activity of potassium nitrate.^12,13^ It has been demonstrated that an increase in the concentration of potassium ions in tissue fluids decreases nerve excitability, which results in the rapid and long-term protection of sensitive teeth.^31^ Fluoride in the tooth structure decreases enamel solubility in acidic environments, prevents dental caries, and strengthens tooth enamel.^32^ Da Rocha Leódido et al^33^ reported that the shear bond strength values of orthodontic brackets after using sodium fluoride solution was 8.66 ± 3.78 MPa, which was acceptable for the shear bond strength of orthodontic brackets. Bishara et al^9^ showed that the shear bond strength was 12 ± 9.5 MPa after using a prophylactic paste, which was not different from the control group. da Silva Fidalgo et al^10^ reported that local fluoride increased the shear bond strength of resin cements. In addition, Whang and Shin^34^ reported that the effect of alpha-tocopherol (a type of vitamin E) on the bond strength was similar to the control group. On the other hand, the effects of some alcoholic agents and chemical solvents on the bond strength of brackets during orthodontic treatments have been confirmed.^6,35^ Studies have shown that alcohol in the composition of a mouthwash can decrease the bond strength.^6,30^ Therefore, alcohol-free mouthwashes such as SensiKIN might not negatively affect the bond strength.^14^ Finally, the overall results of previous studies on the effects of the ingredients of SensiKIN mouthwash on the bond strength might confirm the absence of an adverse effect of this mouthwash on bond strength, which explains the lack of a significant difference in the shear bond strength of orthodontic brackets between the case and control groups.

 Since factors such as the adhesive system type and saliva can affect the bond strength of brackets during fixed orthodontic treatment,^22,36^ further comprehensive studies are required on the effect of SensiKIN mouthwash on the shear bond strength of orthodontic brackets by considering factors such as the adhesive system and saliva.

## Conclusion

 The present study on the effect of SensiKIN mouthwash on the shear bond strength of orthodontic brackets on the enamel surface showed that the mean shear bond strengths of the brackets in the SensiKIN and control groups were 11.8 N (36–387) (approximately 11.7 MPa) and 121.6 N (40.3–473.3) (approximately 12.1 MPa), respectively, with no significant difference between the two groups (*P* = 0.914). Finally, based on the results, the bond strength after using the SensiKIN mouthwash was clinically higher than the minimum bond strength value. This mouthwash did not decrease the shear bond strength of orthodontic brackets. Therefore, it can be used during orthodontic treatment.

## Competing Interests

 The authors declare no conflicts of interest with regard to authorship and/or publications of this paper.

## Ethical Approval

 The study protocol was approved by the Ethics Committee at Tabriz University of Medical Sciences under the code IR.TBZMED.REC.1399.351. In this study, patient consent was not applicable.

## References

[1] Tsichlaki A, Chin SY, Pandis N, Fleming PS (2016). How long does treatment with fixed orthodontic appliances last? A systematic review. Am J Orthod Dentofacial Orthop.

[2] da Rocha JM, Gravina MA, da Silva Campos MJ, Quintão CC, Elias CN, Vitral RW (2014). Shear bond resistance and enamel surface comparison after the bonding and debonding of ceramic and metallic brackets. Dental Press J Orthod.

[3] Park SB, Son WS, Ko CC, García-Godoy F, Park MG, Kim HI (2009). Influence of flowable resins on the shear bond strength of orthodontic brackets. Dent Mater J.

[4] Atassi F, Awartani F (2010). Oral hygiene status among orthodontic patients. J Contemp Dent Pract.

[5] Zachrisson BU (1974). Oral hygiene for orthodontic patients: current concepts and practical advice. Am J Orthod.

[6] Jamilian A, Saghiri MA, Ghasemi M, Ghasemian A, Borna N, Kamali Z (2011). The effects of two mouth rinses on shear bond strength of orthodontic brackets-an in-vitro study. Virtual J Orthod.

[7] Karaman AI, Uysal T (2004). Effectiveness of a hydrophilic primer when different antimicrobial agents are mixed. Angle Orthod.

[8] Cacciafesta V, Sfondrini MF, Stifanelli P, Scribante A, Klersy C (2006). The effect of bleaching on shear bond strength of brackets bonded with a resin-modified glass ionomer. Am J Orthod Dentofacial Orthop.

[9] Bishara SE, Damon PL, Olsen ME, Jakobsen JR (1996). Effect of applying chlorhexidine antibacterial agent on the shear bond strength of orthodontic brackets. Angle Orthod.

[10] da Silva Fidalgo TK, Pithon MM, do Santos RL, de Alencar NA, Abrahão AC, Maia LC (2012). Influence of topical fluoride application on mechanical properties of orthodontic bonding materials under pH cycling. Angle Orthod.

[11] Jamilian A, Saghiri MA, Ghasemi M, Ghasemian A. The effects of ortho kin and oral b mouth rinses on the shear bond strength of orthodontic brackets (in vitro). J Res Dent Sci 2010;7(1):25-33. [Persian].

[12] Kadkhoda Z, Rafiei Chokami S, Hosseini A. Effectiveness of a desensitizing dentifrice containing potassium nitrate 5% on cervical hypersensitivity of teeth: a randomized clinical trial. J Dent Med 2017;30(1):33-9. [Persian].

[13] Moghareh Abed A, Naghsh N, Birang R, Shafiei F, Seifi M, Yaghini J (2012). Clinical evaluation of the efficacy of neodymium-doped yttrium aluminium garnet (Nd:YAG) laser therapy and Sensikin® in treatment of dentine hypersensitivity. J Lasers Med Sci.

[14] Sensikin Mouthwash Brochure. Available from: https://kinprofesional.kin.es/en/sensitivity/sensikin-mouthwash-250-ml-sa-2634.html.

[15] Demir A, Malkoc S, Sengun A, Koyuturk AE, Sener Y (2005). Effects of chlorhexidine and povidone-iodine mouth rinses on the bond strength of an orthodontic composite. Angle Orthod.

[16] Dumbryte I, Linkeviciene L, Malinauskas M, Linkevicius T, Peciuliene V, Tikuisis K (2013). Evaluation of enamel micro-cracks characteristics after removal of metal brackets in adult patients. Eur J Orthod.

[17] Naisbitt MA. The Effect of Bracket Base Design on Shear Bond Strengt - At the Bracket Base-Cement Interface. Birmingham: University of Alabama at Birmingham; 2010. p. 17.

[18] Pignatta LM, Duarte Júnior S, Santos EC (2012). Evaluation of enamel surface after bracket debonding and polishing. Dental Press J Orthod.

[19] Uysal T, Baysal A, Uysal B, Aydınbelge M, Al-Qunaian T (2011). Do fluoride and casein phosphopeptide-amorphous calcium phosphate affect shear bond strength of orthodontic brackets bonded to a demineralized enamel surface?. Angle Orthod.

[20] Ansari MY, Agarwal DK, Gupta A, Bhattacharya P, Ansar J, Bhandari R (2016). Shear bond strength of ceramic brackets with different base designs: comparative in-vitro study. J Clin Diagn Res.

[21] Bishara SE, Soliman MM, Oonsombat C, Laffoon JF, Ajlouni R (2004). The effect of variation in mesh-base design on the shear bond strength of orthodontic brackets. Angle Orthod.

[22] Dar-Odeh N, Shehabi A, Al-Bitar Z, Al-Omari I, Badran S, Al-Omiri M (2011). Oral Candida colonization in patients with fixed orthodontic appliances: the importance of some nutritional and salivary factors. Afr J Microbiol Res.

[23] Premchind TK, Agarwal A, Kumar RR (2019). Role of biofilm and its effects in orthodontic treatment. J Orofac Health Sci.

[24] Erverdi N, Acar A, Işgüden B, Kadir T (2001). Investigation of bacteremia after orthodontic banding and debanding following chlorhexidine mouth wash application. Angle Orthod.

[25] Bauer Faria TR, Furletti-Goes VF, Franzini CM, de Aro AA, de Andrade TA, Sartoratto A (2021). Anti-inflammatory and antimicrobial effects of Zingiber officinale mouthwash on patients with fixed orthodontic appliances. Am J Orthod Dentofacial Orthop.

[26] Enita N, Dzemidzic V, Tiro A, Hadzic S (2011). Antimicrobial activity of chlorhexidine in patients with fixed orthodontic appliances. Braz J Oral Sci.

[27] Nasir N, Ali S, Bashir U, Ullah A (2011). Effect of orthodontic treatment on periodontal health. Pak Oral Dent J.

[28] Javanmardi Z, Salehi P (2016). Effects of Orthokin, Sensikin and Persica mouth rinses on the force degradation of elastic chains and NiTi coil springs. J Dent Res Dent Clin Dent Prospects.

[29] Catalbas B, Ercan E, Erdemir A, Gelgor IE, Zorba YO (2009). Effects of different chlorhexidine formulations on shear bond strengths of orthodontic brackets. Angle Orthod.

[30] Meeran NA, George AM (2013). Effect of various commercially available mouthrinses on shear bond strength of orthodontic metal brackets: an in vitro study. Indian J Dent Res.

[31] Orchardson R, Gillam DG (2006). Managing dentin hypersensitivity. J Am Dent Assoc.

[32] Sudjalim TR, Woods MG, Manton DJ, Reynolds EC. Prevention of demineralization around orthodontic brackets in vitro. Am J Orthod Dentofacial Orthop 2007;131(6):705.e1-705.e9. 10.1016/j.ajodo.2006.09.043. 17561043

[33] da Rocha Leódido G, Fernandes HO, Tonetto MR, Presoto CD, Bandéca MC, Firoozmand LM (2012). Effect of fluoride solutions on the shear bond strength of orthodontic brackets. Braz Dent J.

[34] Whang HJ, Shin DH (2015). Effects of applying antioxidants on bond strength of bleached bovine dentin. Restor Dent Endod.

[35] Oncag G, Tuncer AV, Tosun YS (2005). Acidic soft drinks effects on the shear bond strength of orthodontic brackets and a scanning electron microscopy evaluation of the enamel. Angle Orthod.

[36] Sung EC, Chan SM, Tai ET, Caputo AA (2004). Effects of various irrigation solutions on microleakage of class V composite restorations. J Prosthet Dent.

